# iTRAQ based protein profile analysis revealed key proteins involved in regulation of drought-tolerance during seed germination in Adzuki bean

**DOI:** 10.1038/s41598-021-03178-y

**Published:** 2021-12-09

**Authors:** Xuesong Han, Fangwen Yang, Yongguo Zhao, Hongwei Chen, Zhenghuang Wan, Li Li, Longqing Sun, Liangjun Liu, Chunhai Jiao, Changyan Liu, Aihua Sha

**Affiliations:** 1 Institute of Food Crops, Hubei Academy of Agricultural Sciences/Hubei Key Laboratory of Food Crop Germplasm and Genetic, Wuhan, China; 2 Shanghai Agro-Biological Gene Center, Shanghai, China; 3 Guangdong University of Petrochemical Technology, Maoming, China; 4 College of Agronomy, Yangtze University, Jingzhou, China

**Keywords:** Molecular biology, Plant sciences

## Abstract

Adzuki bean is an important legume crop due to its high-quality protein, fiber, vitamins, minerals as well as rich bioactive substances. However, it is vulnerable to drought at the germination stage. However, little information is available about the genetic control of drought tolerance during seed germination in adzuki bean. In this study, some differential expression proteins (DEPs) were identified during seed germination between the drought-tolerant variety 17235 and drought-sensitive variety 17033 in adzuki bean using iTRAQ method. A total of 2834 proteins were identified in the germinating seeds of these two adzuki beans. Compared with the variety 17033, 87 and 80 DEPs were increased and decreased accumulation in variety 17235 under drought, respectively. Meanwhile, in the control group, a few DEPs, including 9 up-regulated and 21 down-regulated proteins, were detected in variety 17235, respectively. GO, KEGG, and PPI analysis revealed that the DEPs related to carbohydrate metabolism and energy production were significantly increased in response to drought stresses. To validate the proteomic function, the ectopic overexpression of V-ATPase in tobacco was performed and the result showed that V-ATPase upregulation could enhance the drought tolerance of tobacco. The results provide valuable insights into genetic response to drought stress in adzuki bean, and the DEPs could be applied to develop biomarkers related to drought tolerant in adzuki bean breeding projects.

## Introduction

Drought is one of most devastating environmental stress that decreases crop productivity^1^. The occurrence of drought will be more frequent as the global temperature is increasing and the fresh water is lacking^2^. Drought stress impact more seriously on germinating seeds and seedling development phases in most crops, which results in the delaying of seed germination and reduction of the germination rate at a very early developmental stage^3^. The establishment, growth, and productivity of crop is required for a high rate and uniformity of germination under drought. Therefore, understanding the genetic basis involved in seed germination under drought is helpful for further increasing yield potential^3^.

Seed germination is accomplished by a well-orchestrated series of events such as phytohormones and other small molecules mediated interactions with the environment, which signal a suitable environment for germination to ensure plant survival^4^. Specifically, ABA and GAs are considered to be indispensable for seed germination, and their dynamic equilibrium is central to the control of seed dormancy and germination^5^. Drought stress can delay or prevent the seed germination through reducing water availability, changing the mobilization of stored reserves, and affecting the structural organization of proteins^6^. To adapt to drought stress, plants developed the concerted mechanisms such as mechano-receptors, ion transport channels, and secondary signal molecules to maintain ion homeostasis as well as cascades of gene activations for hormonal metabolism, signal transduction pathways, and stress responses^7^.

Adzuki bean (*Vigna angularis* L.) belongs to the *Vigna* of *Leguminosae*^8^. It is the second-most important legume crop behind soybean and is widely grown in East Asian countries such as China, Japan, and Korea^9^. Adzuki bean is favored by Asian consumers due to its high-quality protein, fiber, vitamins, minerals as well as rich bioactive substances^10,11^. Although adzuki bean is often planted in dry land as a paddy crop^12^, it is vulnerable to drought at the germination stage^13^. Therefore, it is significant to improve its drought resistance to obtain the ideal productivity in arid environments. Understanding the genetics and exploiting key genes controlling drought tolerance at the germination stage of adzuki bean will be beneficial to create drought tolerant adzuki bean.

Recently, a number of genes, QTLs, or proteins in germinating seed responding to drought were identified in some crops. A genome-wide association study (GWAS) identified 338 single nucleotide polymorphisms (SNPs) that were associated with seed germination-related traits under drought in barley^3^. Ten Quantitative trait loci (QTL) were detected to contribute to germination or early seedling drought tolerance in the interspecific cross *Setaria italic* × *Setaria viridis*^14^. Thirty-nine QTLs were identified in response to drought stress during seed germination in *Brassica napus*, in which 256 candidate genes were obtained by co-linear analysis between genetic and physical maps^15^. A total of 1200 proteins were detected to differentially accumulate under drought stress in maize by proteomics analysis^16^.

To date, little information is available about the genetic responses of drought tolerance during seed germination in adzuki bean. Several bZIP genes were reported in response to drought and salt stress in *Vigna radiata* and *V. angularis* by whole-genome sequencing and quantitative real-time PCR analysis^17^. We previously selected a drought-tolerant variety and a drought-sensitive variety through the drought tolerance identification of adzuki bean seeds, and identified 82 differentially expressed genes (DEGs) in the germinating seeds between drought tolerant and drought sensitive variety of adzuki bean under drought stress by PacBio SMRT and Illumina Sequencing^18^. It provided a ideal materials for investigating protein expression patterns of two varieties under drought stress. In this study, the proteomics approach was adopted to further identify the differentially expressed proteins (DEPs) in germinating seed of adzuki bean by comparing the drought-tolerant variety (No. 17235) and drought-sensitive variety (No. 17033). The proteins related to carbohydrate metabolism and energy production were significantly upregulated in response to drought stress. The findings are helpful to understand the genetic regulation of drought stress during seed germination, and provide a foundation for breeding drought-tolerant adzuki varieties in future breeding projects.

## Result

### Quantitative proteomic analysis

The previously identified drought-tolerant varietyNo. 17235 and drought sensitive varietyNo. 17033^13^, were used to conduct the proteomic analysis in this study. The seeds of No. 17235 and No. 17033 were treated with mannitol (MA) solution (named as 17235T and 17033T, respectively) and the deionized water as control groups (named as 17235CK and 17033CK, respectively). The seeds were collected after 24 h treatment to analyze protein profiles using the iTRAQ technology. A total of 179,175 spectra were obtained after eliminating the low-scoring spectra. Finally, 14,563 peptides, 10,764 unique peptides, and 2,834 protein groups were identified. The molecular weights of 93.33% of the identified proteins were up to 10 kDa. The mass deviation of all identified peptides was mainly distributed within 10 ppm with the ideal score of MASCOT. The scores of about 80% peptides were beyond 20, and the abundance ratios of most proteins in the two groups of equal-labeled samples is close to 1 (Tables S1, S2).

### Identification of differentially expressed proteins

Drought-responsive proteins were identified by the pairwise comparison of protein abundance between control and MA-treated samples using the iTRAQ data. The criterion of fold change ratio > 1.20 or < 0.83 and *p* < 0.05 was used to identify differentially expressed proteins (DEPs) between the treatments and control groups.

**Figure 1:**
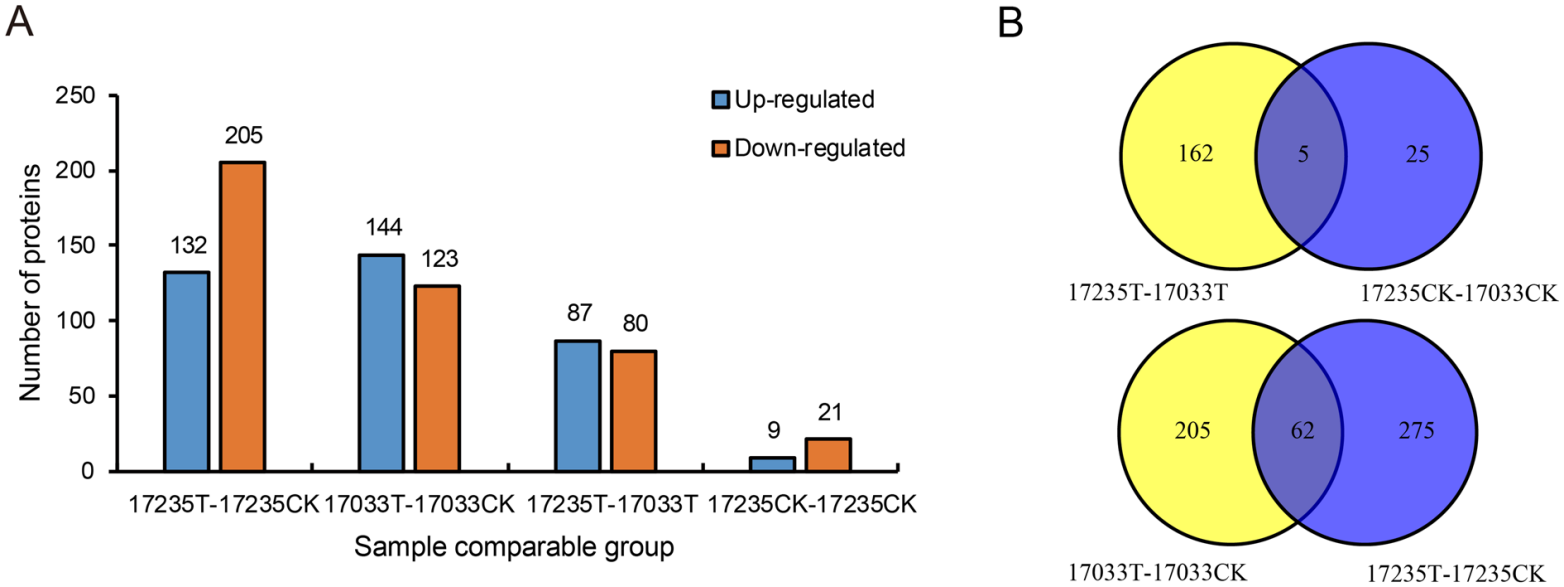
Quantitative and Venn analysis of the proteome of two adzuki bean under different treatments. (A) Quantitative analysis of the proteome between the MA treated and control samples; (B) Venn analysis of two adzuki bean under different treatments.

To investigate the candidate proteins that play roles on the different tolerance of the two varieties, we firstly focus on the DEPs identified with 17235T and 17033T. A total of 167 DEPs were identified, in which 87 were up-regulated and 80 down-regulated, respectively (Fig. 1A, Table S3). In the control groups, 30 DEPs were identified between 17235 and 17033CK (Fig. 1A, Table S3). With drought stress, the different protein expression levels in these two varieties could reflect their different abilities of drought tolerance. Notably, 5 DEPs were identified in the two comparisons of 17235T versus 17033T and 17235CK versus 17033CK (Fig. 1B, Table S3), implying these DEPs might be variety-specific proteins. To investigate drought-responsive proteins in different varieties, we analyzed the DEPs in the comparisons of 17235T versus 17235CK and 17033T versus 17033CK. A total of 337 and 267 DEPs were identified in the comparisons of 17235T versus 17235CK and 17033T versus 17033CK, respectively. Among them, 132 up-regulated DEPs and 205 down-regulated DEPs were detected in comparisons of 17235T versus 17235CK. Likewise, 144 DEPs were up-regulated and 123 DEPs were down-regulated in comparisons of 17033T versus 17033CK (Fig. 1A, Table S3). In addition, 62 DEPs were detected in the two comparisons of 17235T versus 17235CK and 17033T versus 17033CK, suggesting that these DEPs might be drought responding proteins.

### Functional annotation of drought-responsive proteins

**Figure 2:**
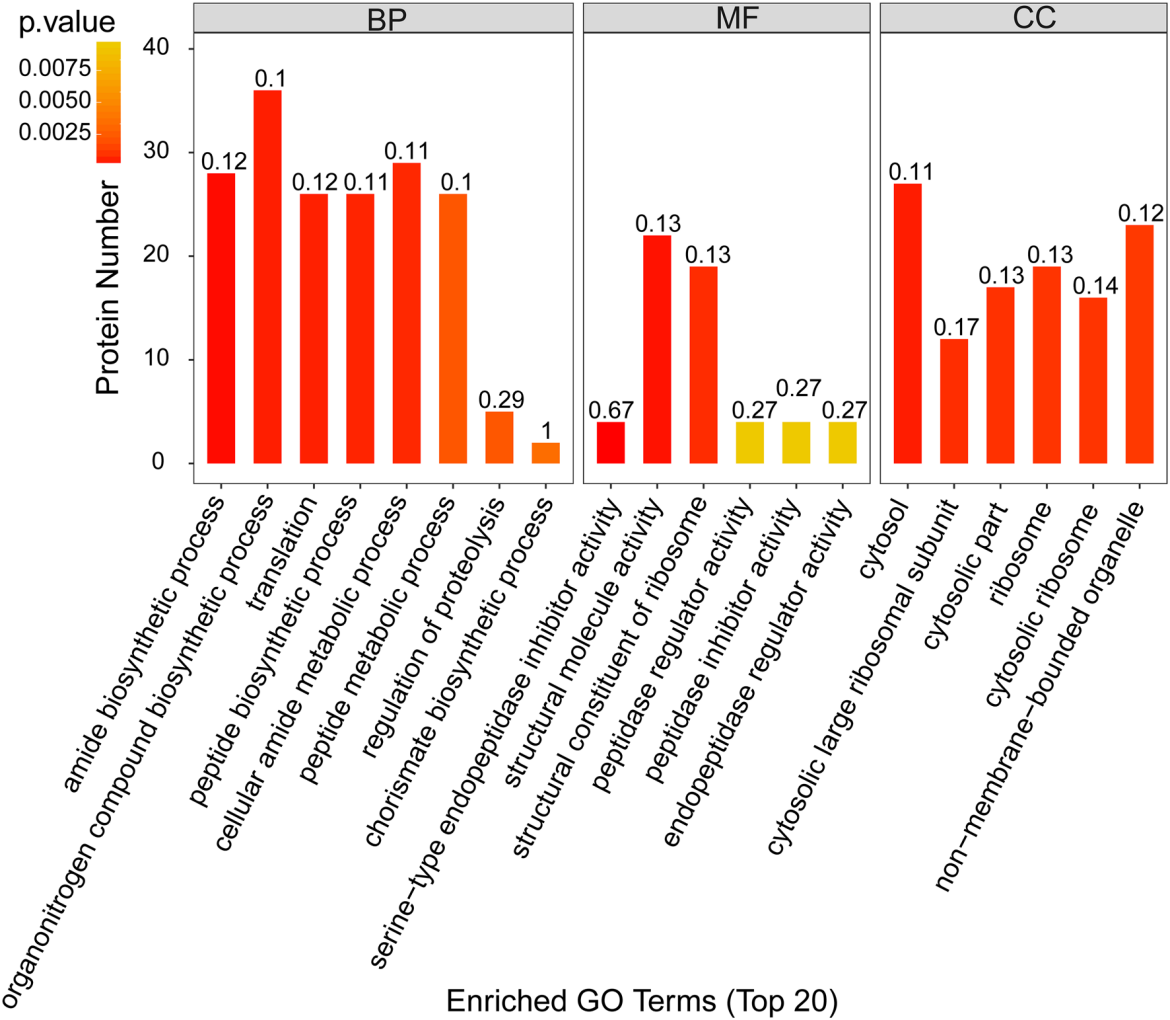
GO enrichment annotation of differentially expressed proteins between 17235 and 17033 under drought stress. The abscissa in the figure represents the enriched GO functional classification, BP (Biological Process,), MF (Molecular Function) and CC (Cellular Component) three categories; the ordinate indicates the number of differential proteins under each functional classification. The color of the bar graph indicates the importance of rich GO function classification, which is the P value calculated based on Fisher's exact test. The label above the bar graph displays richFator (richFator <  = 1), which represents the proportion of the number of differentially expressed proteins annotated to a GO functional category to the number of all identified proteins annotated to the GO functional category.

The DEPs in the comparison of 17235T versus 17033T belonged to 111 biological processes, 101 cellular compartments, and 125 molecular functions, respectively (Table S4). In terms of biological process, metabolic process and cellular process were the major groups. Catalytic activity and binding were the top two major molecular functional groups. Cell and cell part were the top two cellular compartments. “structural molecule activity”, “structural constituent of ribosome” were enriched by DEPs in the category of molecular function, indicating that structural molecule activity play a major role in regulating drought tolerance between different varieties; In the category of cellular component, the GO terms related with several cytosolic proteins were detected, such as “cytosolic large ribosomal subunit”, “cytosolic part”, “cytosol”, “cytosolic ribosome” (Fig. 2). These cytosolic proteins may be involved in the regulation of seed permeation to regulate drought tolerance in germinating seeds.

In addition, GO enrichment analysis was performed with the DEPs from the comparisons of 17235T versus 17235CK and 17033T versus 17033CK. In Cellular components, cytoplasm, intracellular part and intracellular were significantly enriched. Peptide metabolic process, cellular amide metabolic process and translation are significantly enriched in biological process. In molecular function. structural molecule activity, guanyl nucleotide binding and GTP binding are significantly enriched (Table s4).

KEGG annotation indicated that the DEPs in comparison of 17235T versus 17033T were assigned to 110 KEGG pathways. Among them, 4 pathway categories, including Ribosome, Phagosome, Fructose and mannose metabolism, and Insulin signaling pathway, were significantly enriched (Table S5). In the comparison of 17235T versus 17235CK, 15 pathways were significantly enriched. Among them, 5 pathways are related to regulation of drought stress, including Ribosome, Peroxisome, Glycolysis / Gluconeogenesis, and Starch and sucrose metabolism as well as Endocytosis (Table S5).

### Protein–protein interaction among DEPs

**Figure 3:**
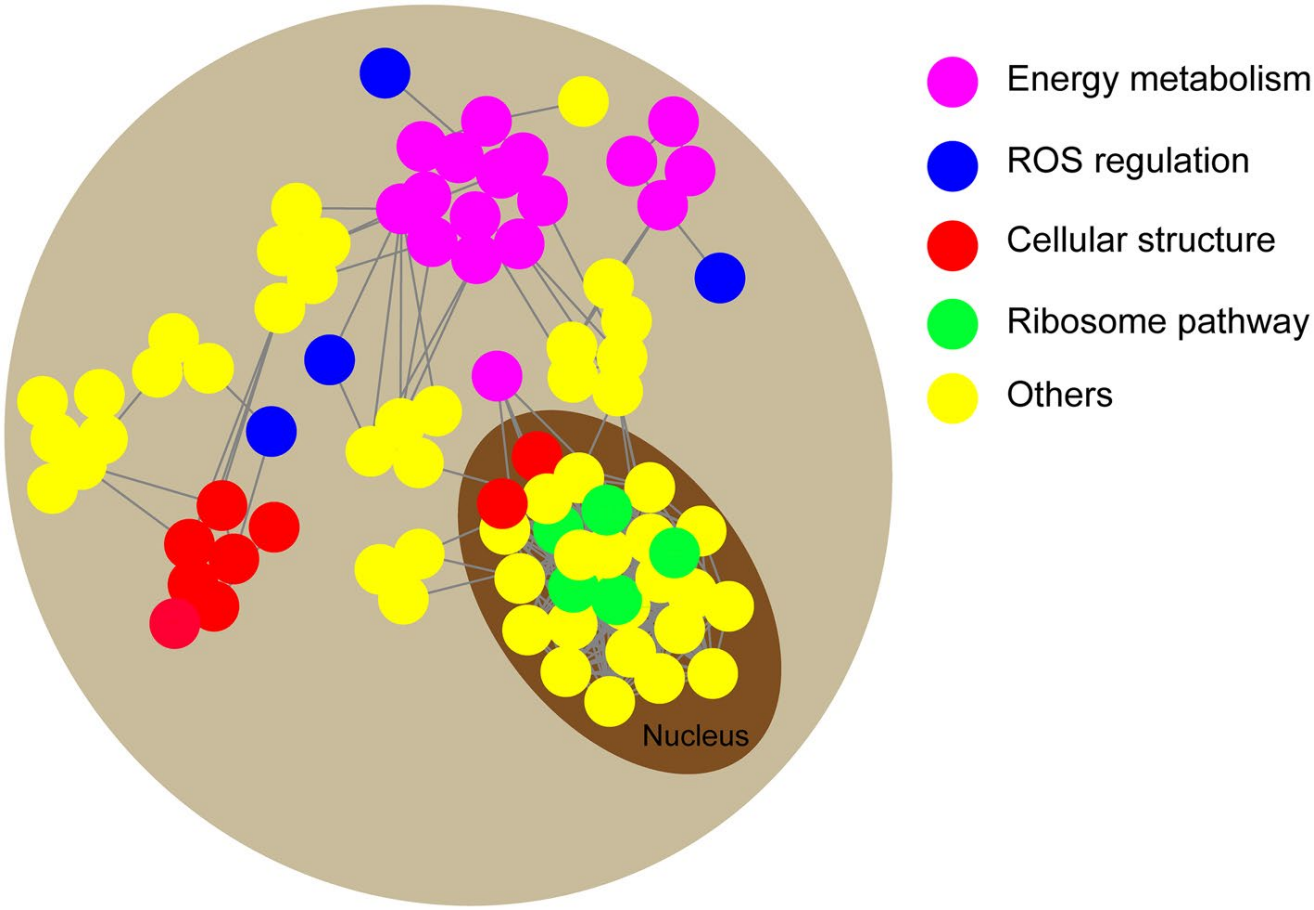
PPI network of differential protein under 17235T versus 17033T. The Purple cycle are energy metabolism proteins, blue cycle are ROS regulation proteins, red cycle are cellular structure proteins, green cycle are ribosome pathway proteins, yellow cycle are others proteins, and the protein in the dark brown ellipse is the protein predicted to be in the nucleus.

To predict the relationship among all these DEPs identified in adzuki bean, a protein–protein interaction (PPI) network was generated using the web-tool STRING 9.1. A total of 167 DEPs represented by 99 unique proteins from adzuki bean were shown in the PPI network (Fig. 3, Table S6). Four functional modules forming tightly-connected clusters were illuminated in the network. Nodes in different colors belong to the major metabolic pathways. In module RNA-binding Proteins, a large number of ribosomal proteins are closely linked to the RNA-binding proteins and transport RNA. It indicated that the genes in ribosome pathway may play key roles in the early germination of adzuki bean seeds under drought stress. Plant cytoskeleton related proteins connected together in module cellular structure, indicating that these genes related to cell structure may mediate seed germination under drought stress. The module of energy metabolism included the proteins related to multiple enzymes involved in the TCA cycle, glycolysis, amino acid metabolism and nitrogen metabolism. These linked proteins also indicated that a synergistic system for carbon and nitrogen metabolism may play important roles in drought response. The distribution of ROS regulation module is relatively scattered, indicating that ROS is produced in different metabolic processes, but it is related to energy metabolism, which also shows that the process of energy metabolism is closely related to the production and regulation of ROS.

In addition, PPI analysis was performed on the differential proteins in the comparison of 17235CK versus 17033CK. A total of 29 DEPs represented by 14 unique proteins from adzuki bean were shown in the PPI network (Fig. S1, Table S6). These proteins are mainly related to RNA binding and protein translation modification, which is also the difference in the germination period between the two varieties under normal conditions.

### qRT-PCR assay

**Figure 4:**
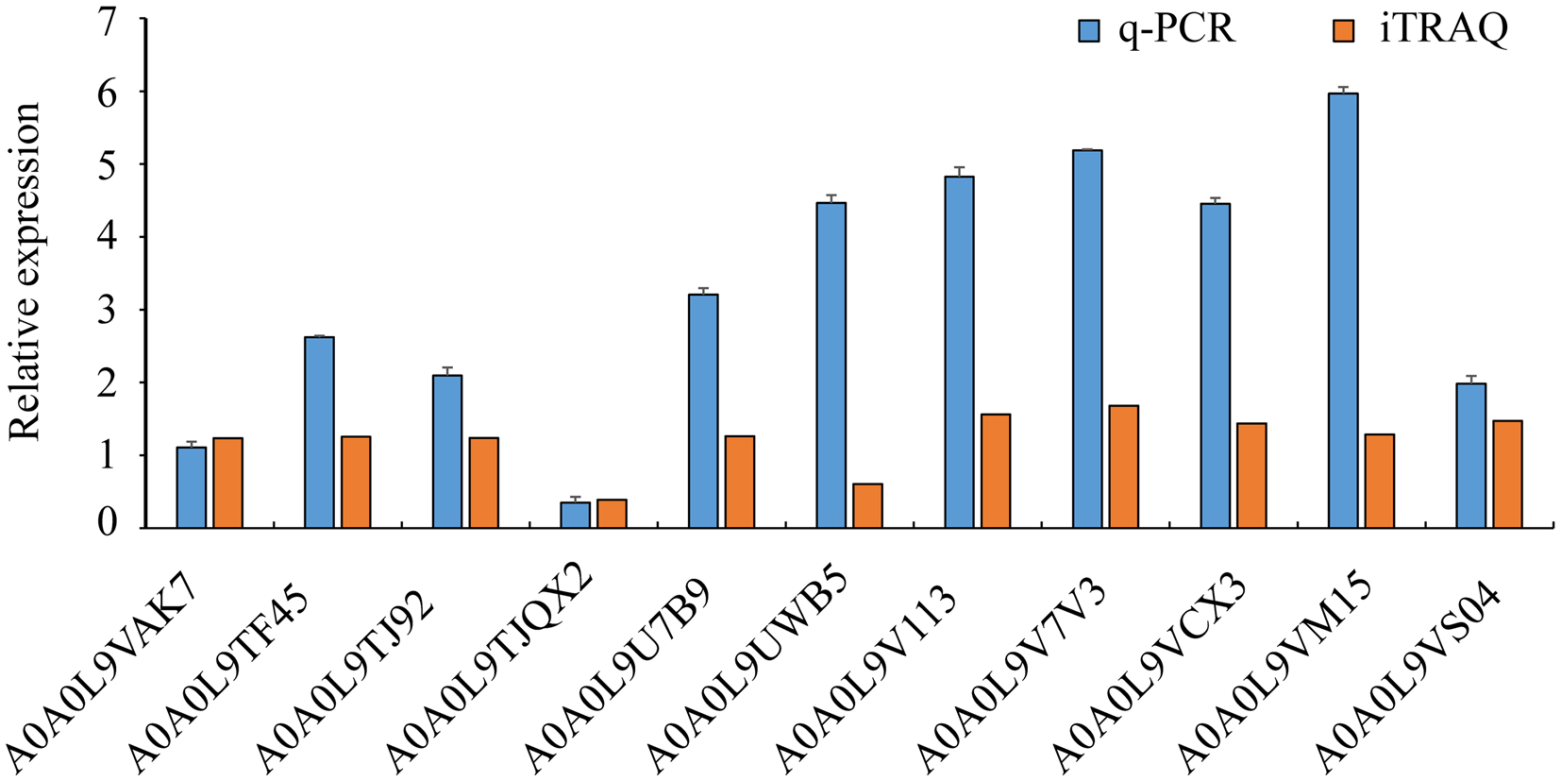
Differential protein quantification at transcript and translation level. Differences in protein expression and qRT-PCR between 17235 and 17033 under drought stress. q-PCR represents RNA expression level; iTRAQ represents the differences in protein expression level.

To investigate transcription patterns, we used the samples of 17235T and 17033T as ITRAQ proteome to extract RNA, and reversely transcribed for q-PCR. A total of 11 proteins identified by iTRAQ were used for qRT-PCR analyses. Some of these DEPs have been reported to be involved in stress response. The fold changes of protein are shown in Table S3, and the primers for qRT-PCR are listed in Table S7. The internal reference gene actin was used to normalize the transcriptional level of target genes in the calculation of 2^−ΔΔCT^. The qRT-PCR results indicated that the gene expression levels of 11 DEPs (A0A0L9VAK7, A0A0L9TF45, A0A0L9TJ92, A0A0L9TQX2, A0A0L9U7B9, A0A0L9UWB5, A0A0L9V113, A0A0L9V7V3, A0A0L9VCX3, A0A0L9VM15, A0A0L9VS04) were consistent with the iTRAQ results (Fig. 4).

### Functional verification of DEP

**Figure 5:**
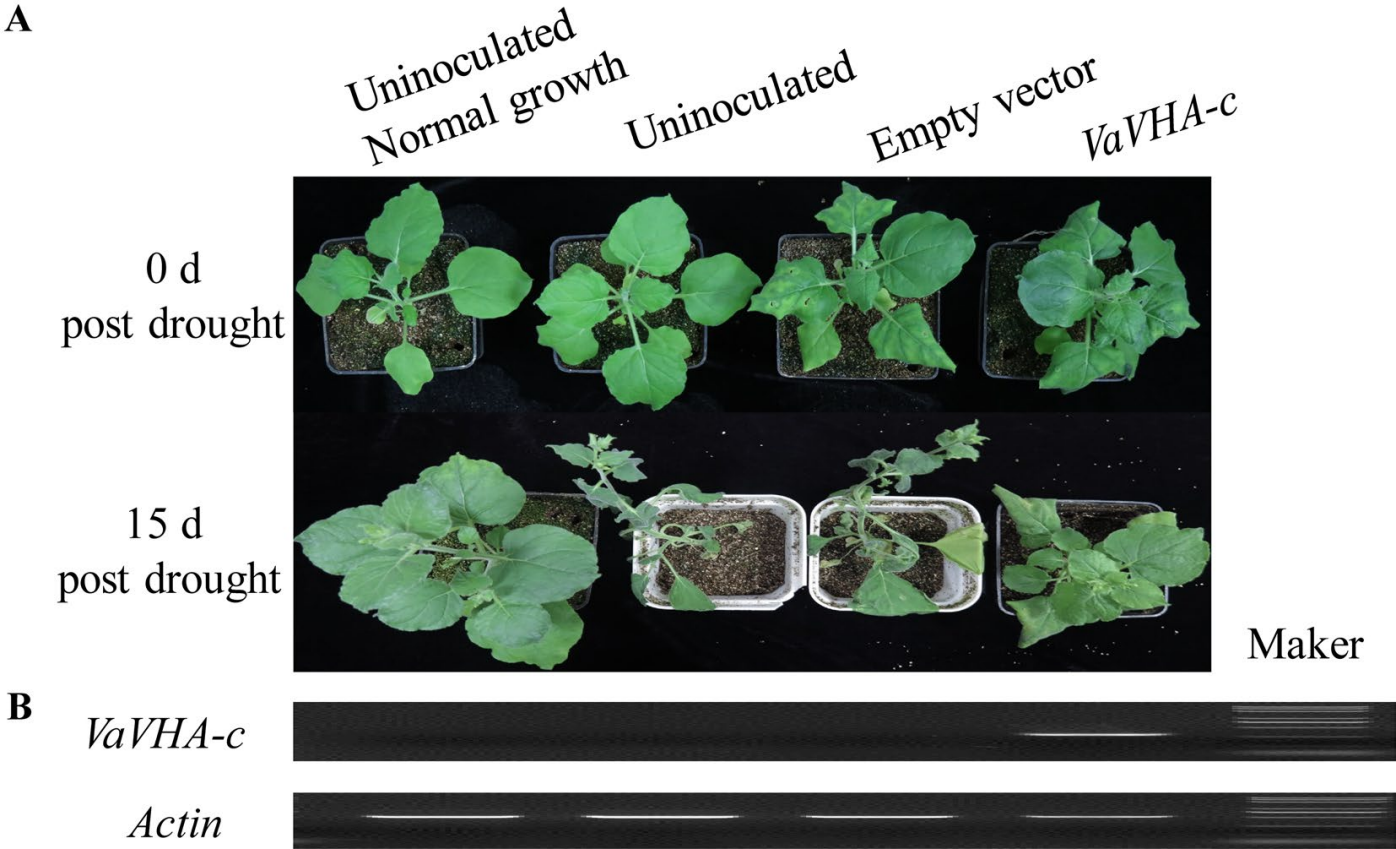
Function validation of* VaVHA-c*. (**A**) phenotype of plants with overexpressed *VaVHA-c*, empty PVX vector, or uninoculated growth under normal condition (0 d post drought) or drought stress condition (15 d post drought). (**B**) expression of *VaVHA-c *detected by RT-PCR (the original gel blot seen in Fig. S2).

**Figure 6:**
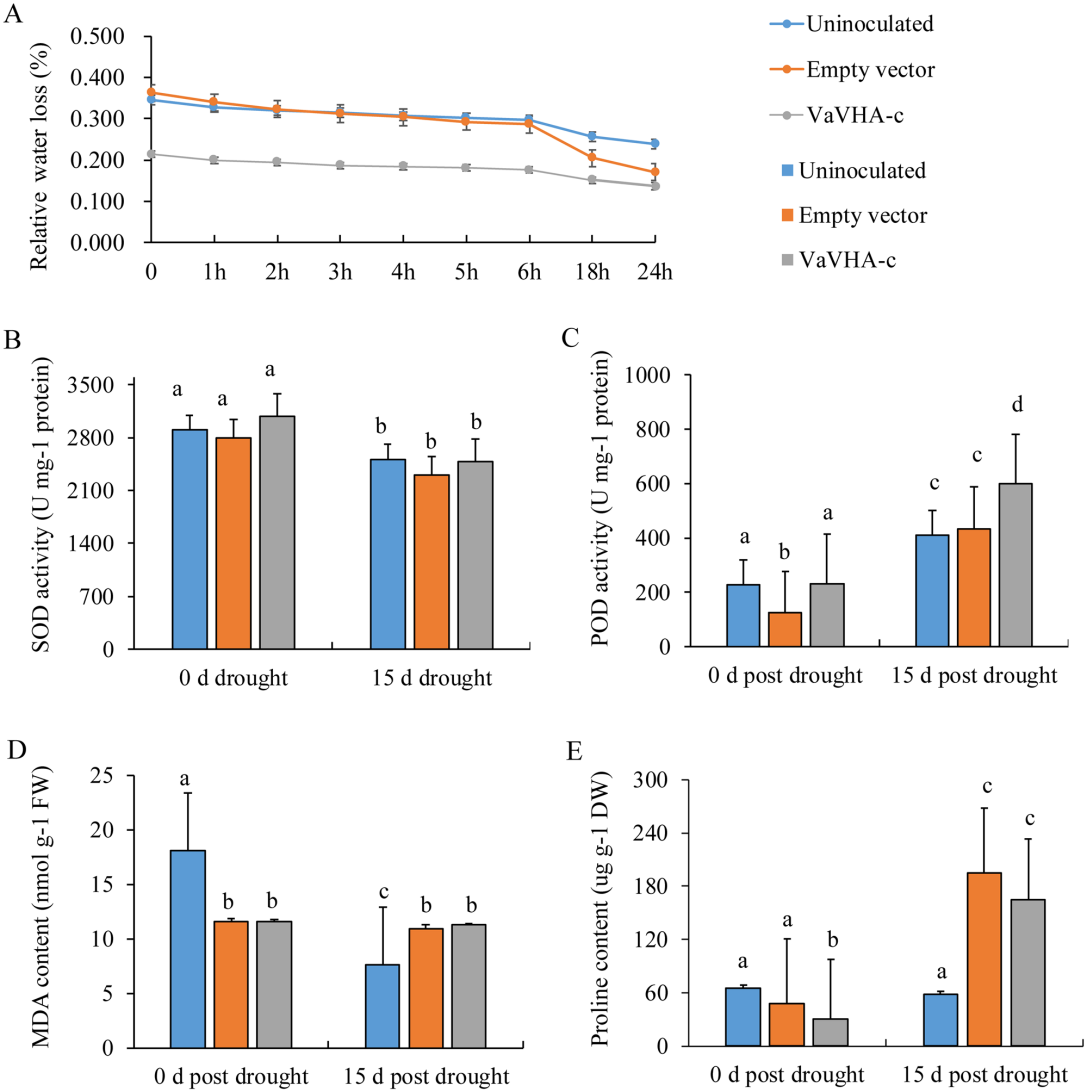
Physiological analysis of *VaVHA-c* ectopic expressed tobacco under drought. (**A**) Water loss; (**B**,**C**) Activity of SOD and POD, respectively; (**D**,**E**) Content of MDA and proline, respectively. DW, dry weight; FW, Fresh weight; MDA, malondialdehyde; SOD: superoxide dismutase; POD: peroxidase. The lowercase letters represent significant difference (*P* < 0.05) between ectopic expressed lines and control plants. Values are means ± SE of three replicates.

To adapt to drought stress, plants developed the concerted mechanisms such as mechano-receptors, ion transport channels, and secondary signal molecules to maintain ion homeostasis as well as cascades of gene activations for hormonal metabolism, signal transduction pathways, and stress responses^7^. PVX based vector can be used to mediate high expression of foreign genes, and the overexpressed protein can move away from the initially infected cells to the uninfected sites^19,20^. Virus-based vectors were used to investigate gene function involved in drought stress^21,22^. In this study, V-ATPase (A0A0L9TJ92) was up-regulated in 17235T compared to 17033T, and its expression levels at transcriptomic and proteomic level were consistent. To verify whether this protein can improve the drought tolerant in plant, the full-length cDNA of *VaVHA-c* was amplified from adzuki bean seeds and was ectopic overexpressed in *Nicotiana benthamiana* by the PVX virus vector. The ectopic overexpression of *VaVHA-c* obviously enhanced the drought tolerance in tobacco. The new developping leaves that was unincoculated in plants with ectopic overexpression of *VaVHA-c* grew normally, however, the plants in control group inoculated with empty vector wilted after 15 d drought treatment (Fig. 5A). The expression of *VaVHA-c* could be detected only in the ectopic overexpression of *VaVHA-c* plants by RT-PCR (Fig. 5B), implying that the enhanced drought tolerance was due to the overexpression of *VaVHA-c*. Measurements of physiological parameters indicated that the activity of peroxidase (POD) was increased, and water loss was reduced in the leaves of plants with overexpressed *VaVHA-c* under drought (Fig. 6).

## Discussion

Germination is a complex process involving events associated with the transition of a quiescent dry seed to a metabolically active state^23,24^. The germination efficiency determines the seedling establishment and the proper development of mature plants. In the study, a number of DEPs were detected between the drought-tolerant and drought-sensitive varieties (Fig. 1), and 4 functional modules energy metabolism, cellular structure, ROS regulation, RNA-binding proteins were formed tightly-connected clusters in PPI network in this study (Fig. 3), implying that DEPs involved in these processes might played the key roles in regulatory of germination under stress.

**Figure 7:**
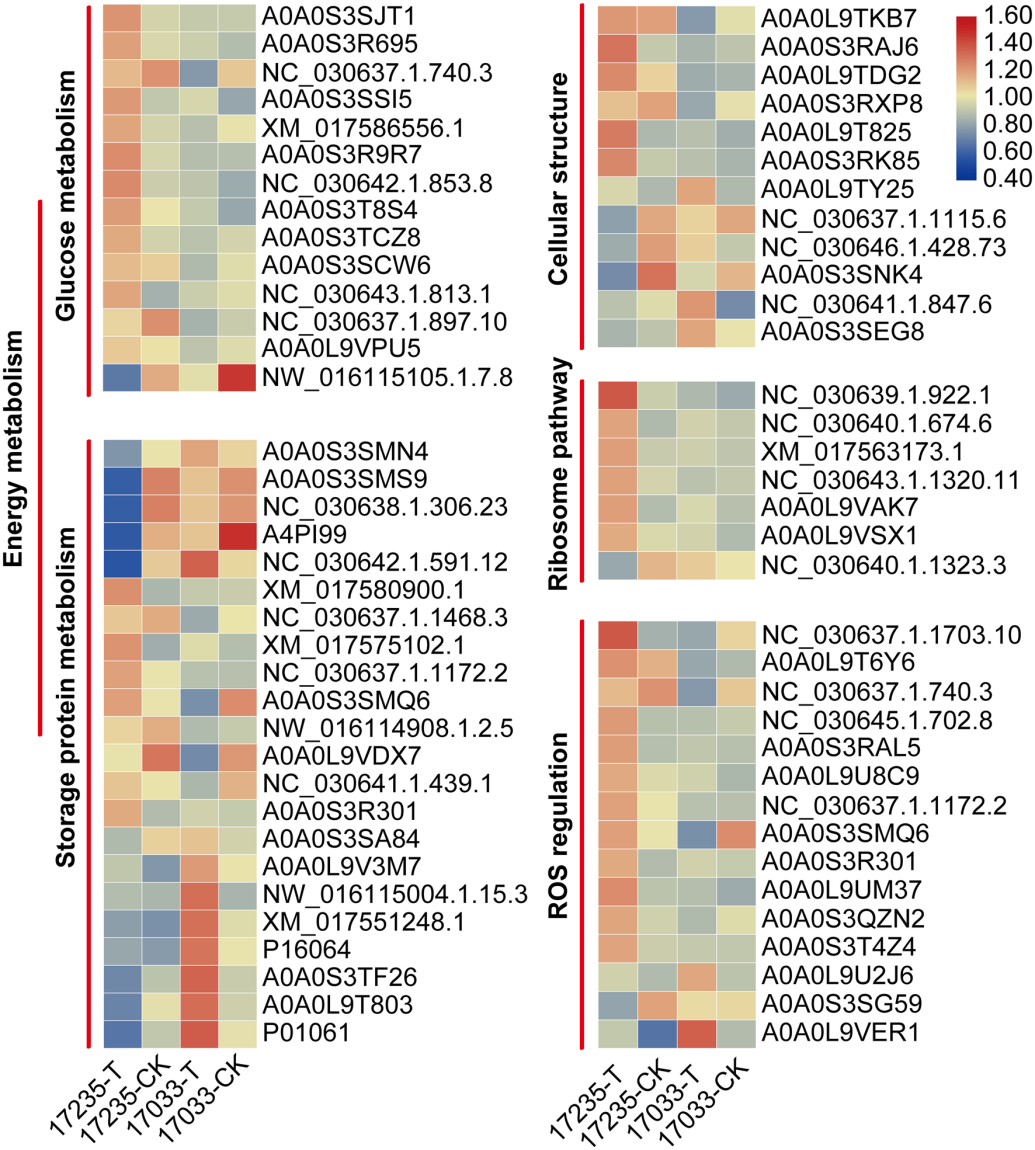
Heat map of four functionally grouped differential proteins.

The cytoskeleton-associated proteins have been determined to be key regulatory molecules in mediating cytoskeleton reorganization in response to multiple environmental signals, such as light, salt, drought and biotic stimuli^25^. Reorganization of cellular structure actin is a central component of the cytoskeleton^3^. Actin and the microtubule network are regulated by many factors such as EF1α, Ca^2+^/CaM, tubulin cofactors (TBCC)^26–29^. *OsADF3*-heterologous transgenic *Arabidopsis* increased drought stress tolerance and up-regulated many downstream drought responsive genes^30^. Overexpression of *LreEF1A4* improved seed germination rate under drought stress^31^. In this study, we detected several up-regulated DEPs related to cell structure such as A0A0L9TKB7 (Tubulin alpha chain), A0A0S3RAJ6 (Tubulin beta chain), A0A0L9TDG2 (actin family), A0A0S3RXP8 (TBCC domain-containing protein 1), A0A0L9T825 (T-complex protein 1 subunit gamma), A0A0S3RK85 (T-complex protein 1 subunit eta) (Fig. 7 and Table S8) under drought stress in the drought-tolerant variety.These proteins may provide a vital structural basis for seed germination under drought stress. In addition, the cell wall-related proteins NC_030646.1.428.73, A0A0S3SNK4 (Dirigent protein), NC_030641.1.847.6 (epidermis-specific secreted glycoprotein EP1), A0A0S3SEG8 (expansin) were not consistent with previous studies^32–34^, which may be the specific metabolic activity of adzuki bean under drought conditions.

Energy materials including carbohydrates, proteins and lipids in seeds are utilized as nutrients and energy sources at the germination stage. Starch and proteins are mobilized via the activation of corresponding amylases and proteases^35^. The carbohydrate can be utilized more easily in metabolism due to its simple molecular structure^36^. The enhanced carbohydrate metabolism would accelerate reserve hydrolysis and impair protein biosynthesis during seed germination^37^. In this study, some DEPs were detected in the pathways related with energy and carbohydrate metabolism, such as alpha-1,4 glucan phosphorylase, pyrophosphate-fructose 6-phosphate 1-phosphotransferase, sucrose synthase. Most of these DEPs were up-regulated, and five seed storage proteins A0A0S3SMN4, A0A0S3SMS9, NC_030638.1.306.23, A4PI99, NC_030642.1.591.12 (Fig. 7 and Table S8) were significantly down-regulated in the drought-tolerant variety under drought stress, implying that more energy provided might result in the drought-tolerance variety under drought stress.

In addition, storage proteins must be degraded to sustain embryo growth and development until an autotrophic growth is reached. Several protease families are involved in the germination process^38^. Notably, 9 proteases were up-regulated, and 8 protease inhibitors are down-regulated in the drought-tolerance variety under drought (Fig. 7 and Table S8), suggesting that the seeds of drought-tolerant variety use a large number of proteases to decompose storage proteins to provide energy for their germination and promote seed germination, whereas the sensitive variety seeds decompose more slowly due to protease inhibitors, and inhibit seed germination.

ROS can participate in endosperm weakening during germination through cell wall loosening, but uncontrolled ROS production can lead to oxidative stress and cellular damage, resulting in seed deterioration, preventing germination and early seedling development^39,40^. Some antioxidants such as SOD, POD, glutathione-related enzyme and dehydrogenase was produced to decrease the cellular damage caused by ROS in plants^41^. In this study, several DEPs, including NC_030637.1.1703.10, A0A0L9U8C9, NC_030637.1.740.3, and A0A0S3SSI5, which are involved in regulating ROS homeostasis^42–44^, were detected, suggesting maintenance of ROS homeostasis is important for drought tolerance in adzuki bean seed germination.

V-ATPase (Vacuolar H^+^-ATPases, VHA) regulates the ion balance of cell in plants by pumping H^+^ from the cytosol into the vacuole. V-ATPase was reported to survive plant cell by enhancing its activity under salinity and drought^45,46^. In this study, we found that overexpression of a DEP encoding VaVHA-c could enhance the drought tolerance of plants in *Nicotiana benthamiana*. Furthermore, the POD activity was increased, and the water loss was reduced in the leaves of overexpressed tobacco plants compared to the control group under drought stress. It is suggested that VaVHA-c might participate in maintenance of ROS homeostasis to regulate the tolerance in plant.

## Materials and methods

### Plant growth conditions and treatments

This study is complied with relevant institutional, national, and international guidelines and legislation. The landraces s17235 and s17033 were collected from Hubei Province by Institute of Food Crops, Hubei Academy of Agricultural Sciences, China, which is permitted by government of Hubei Province, and *Nicotiana benthimiana* (*N. benthamiana)* was kept in our lab of Yangtze University, which is permitted to be used for non-commercial purposes.The seeds of s17235 and s17033 were germinated in mannitol (7.5% concentration) and deionized water for 24 h, as described by Zhu et al.^13^. The samples were collected in 24 h, immediately frozen and stored in liquid nitrogen for protein and RNA extraction. Three biological replicates were conducted for each treatment.

### Protein extraction, digestion and iTRAQ labelling

Total proteins were extracted using the cold acetone method. Samples were ground to power in liquid nitrogen, then dissolved in 2 mL lysis buffer (8 M urea, 2% SDS, 1 × Protease Inhibitor Cocktail (Roche Ltd. Basel, Switzerland), followed by sonication on ice for 30 min and centrifugation at 13,000 rpm for 30 min at 4 ℃. The supernatant was transferred to a fresh tube. For each sample, proteins were precipitated with ice-cold acetone at −20 ℃ overnight. The precipitations were cleaned with acetone three times and re-dissolved in 8 M Urea by sonication on ice. Protein quality was examined with SDS-PAGE.

BCA protein assay was used to determine the protein concentration of the supernatant. 100 μg protein per condition was transferred into a new tube and adjusted to a final volume of 100 μL with 8 M Urea. 11 μL of 1 M DTT (DL-Dithiothreitol) was added and samples were incubated at 37 °C for 1 h. Then 120 μL of the 55 mM iodoacetamide was added to the sample and incubated for 20 min protected from light at room temperature.

For each sample, proteins were precipitated with ice-cold acetone, then re-dissolved in 100 μL TEAB. Proteins were then tryptic digested with sequence-grade modified trypsin (Promega, Madison, WI) at 37 °C overnight. The resultant peptide mixture was labeled with iTRAQ tags 113 through 118. The labeled samples were combined and dried in vacuum.

### Strong cation exchange (SCX) fractionation and liquid chromatography–tandem mass spectrometry (LC–MS/MS) analysis

The combined labeled samples were subjected to the SCX fractionation column connected with a high performance liquid chromatography (HPLC) system. The peptide mixture was re-dissolved in the buffer A (buffer A: 20 mM ammonium formate in water, pH 10.0, adjusted with ammonium hydroxide), and then fractionated by high pH separation using Ultimate 3000 system (Thermo Fisher scientific, MA, USA) connected to a reverse phase column (XBridge C18 column, 4.6 mm × 250 mm, 5 μm, (Waters Corporation, MA, USA). High pH separation was performed using a linear gradient starting from 5% B to 45% B in 40 min (B: 20 mM ammonium formate in 80% ACN, pH 10.0, adjusted with ammonium hydroxide). The column was re-equilibrated at initial conditions for 15 min. The column flow rate was maintained at 1 mL/min and column temperature was maintained at 30℃. Twelve fractions were collected; each fraction was dried in a vacuum concentrator for the next step.

Peptide fractions were resuspended with 30 μL solvent C respectively (C: water with 0.1% formic acid; D: ACN with 0.1% formic acid), separated by nanoLC and analyzed by on-line electrospray tandem mass spectrometry. The experiments were performed on an Easy-nLC 1000 system (Thermo Fisher Scientific, MA, USA) connected to a Orbitrap Fusion Tribrid mass spectrometer (Thermo Fisher Scientific, MA, USA) equipped with an online nano-electrospray ion source. 10 μL peptide sample was loaded onto the trap column (Thermo Scientific Acclaim PepMap C18, 100 μm × 2 cm), with a flow of 10 μL/min for 3 min and subsequently separated on the analytical column (Acclaim PepMap C18, 75 μm × 15 cm) with a linear gradient, from 3% D to 32% D in 120 min. The column was re-equilibrated at initial conditions for 10 min. The column flow rate was maintained at 300 nL/min. The electrospray voltage of 2 kV versus the inlet of the mass spectrometer was used.

The fusion mass spectrometer was operated in the data-dependent mode to switch automatically between MS and MS/MS acquisition. Survey full-scan MS spectra (m/z 350–1550) were acquired with a mass resolution of 120 K, followed by sequential high energy collisional dissociation (HCD) MS/MS scans with a resolution of 30 K. The isolation window was set as 1.6 Da. The AGC target was set as 400,000. MS/MS fixed first mass was set at 110. In all cases, one microscan was recorded using dynamic exclusion of 45 s.

### Database search

The mass spectrometry data were transformed into MGF files with Proteome Discovery 1.2 (Thermo, Pittsburgh, PA, USA) and analyzed using Mascot search engine (Matrix Science, London, UK; version 2.3.2). Mascot database was set up for protein identification using *Vigna angularis L* reference genome^47^(ftp://ftp.ncbi.nlm.nih.gov/genomes/all/GCF/001/190/045/GCF_001190045.1_Vigan1.1/), *Vigna angularis L* data in NCBI nr/Swiss-Prot/Uniprot/IPI database, and the PacBio SMRT and illumina sequencing data published by Zhu et al^18^. Mascot was searched with a fragment ion mass tolerance of 0.050 Da and a parent ion tolerance of 10.0 PPM.

### Protein identification and quantification

The Mascot search results were averaged using medians and quantified. Proteins with fold change in a comparison > 1.2 or < 0.83 and unadjusted significance level *p* < 0.05 were considered differentially expressed.

### GO enrichment analysis

GO enrichment analysis provides all GO terms that significantly enriched in DEPs comparing to the genome background, and filter the DEGs that correspond to biological functions. Firstly, all DEPs were mapped to GO terms in the Gene Ontology database (http://www.geneontology.org/), gene numbers were calculated for every term, significantly enriched GO terms in DEGs comparing to the genome background were defined by hypergeometric test. The calculated p-value was adjusted with FDR correction, setting FDR ≤ 0.05 as a threshold. GO terms meeting this condition were defined as significantly enriched GO terms in DEPs. This analysis was able to recognize the main biological functions that DEPs exercise.

### Pathway enrichment analysis

Pathway-based analysis was conducted by blasting against for KEGG database (https://www.kegg.jp/kegg/kegg1.html)48-50. The significantly enriched metabolic pathways or signal transduction pathways in DEPs were identified by comparing with the whole genome background. The calculated p-value was gone through FDR Correction, taking FDR ≤ 0.05 as a threshold. Pathways meeting this condition were defined as significantly enriched pathways.

### RNA extraction and qRT-PCR

Total RNA was extracted using the TRIZOL reagent (Invitrogen, Carlsbad, CA, USA), and then treated with RNasefree DNase (Invitrogen, Gaithersburg, MD, USA). The purified RNA was reverse transcribed using the RevertAid™ First Strand cDNA Synthesis Kit (Thermo Fisher Scientific) according to the manufacturer’s protocol. The qRT-PCR reactions were performed in CFX96™ Real-Time PCR Detection System (Bio-Rad, USA). The gene specific primers were listed in additional Table S7. An actin gene (X69885) of adzuki bean was used as internal control. Each reaction was conducted in 10 μL mixture containing 5 μL of SYBR green (SYBR^@^ Premix Ex Taq™ (TliRNaseH Plus), TAKARA, Japan), 0.3 μL forward and reverse primers (10 μM), respectively, 2 μL cDNA template, and 2.4 μL ddH_2_O. The reactions for each gene were conducted in triplicate with the thermal cycling conditions as follows: 95 °C for 30 s, followed by 40 cycles of 95 °C for 5 s and 57 °C for 30 s. The primer specificity was confirmed by melting curve analysis. Relative expression levels of the genes were calculated using the 2^−ΔΔCT^ method^51^.

### Ectopic expression of VaVHA-c and RT-PCR

Four weeks old *N. benthamiana* plants grown in a growth room (24 °C, 16 h/8 h light/dark, 100 μM m^−2^ s^−1^ white light) were used for *VaVHA-c* ectopic expression. The coding sequence (CDS) region of *VaVHA-c* was amplified with gene specific primers and was inserted into PVX-LIC vector as described by Zhao et al.^52^, and confirmed by sequencing. The resultant construct *VaVHA-c*- PVX-LIC was introduced into *Agrobacterium tumefaciens* GV3101 via the freeze–thaw method, and then introduced into *N. benthimiana* by infiltration method^52^. In brief, PVX derivatives carried GV3101 was cultured, harvested, re-suspended in infiltration buffer, and infiltrated into leaves of *N. benthamiana* through needleless syringes. The empty vector PVX-LIC was introduced into tobacco as negative control. The experiment was performed three times with at least 5 plants for each construct. After 7 d of infiltration, the new developping leaves that was uninoculated were harvested for RNA extraction and RT-PCR analysis, and the plants were treated by withholding water. The phenotype of plants was photographed at 15 d after water withholding. Gene-specific primers were used for RT-PCR and *actin* was used to normalize the reaction as described by Sha et al^51^.

### Physiological parameter measurements and statistical analysis

The new developping leaves that was uninoculated with 10 d water withholding were harvested for analyzing the activity of SOD, POD, content of proline and MDA, and water loss as described by Zhou et al^53^. Data analysis were conducted by Microsoft Excel 2016 and the SPSS 16.0. The significance was analyzed by one-way ANOVA test. Tukey multiple comparison test was used to compared differences at 0.05 significance level.

## Supplementary Information

Supplementary Information 1.

Supplementary Information 2.

Supplementary Information 3.

Supplementary Information 4.

Supplementary Information 5.

Supplementary Information 6.

Supplementary Information 7.

Supplementary Information 8.

Supplementary Information 9.

Supplementary Information 10.

## Data Availability

All data generated or analyzed during this study are included in this published article and its supplementary information files.
