# Supplementary figures and images for: iTRAQ based protein profile analysis revealed key proteins involved in regulation of drought-tolerance during seed germination in Adzuki bean
Source: Sci Rep. 2021 Dec 9;11:23725. doi: 10.1038/s41598-021-03178-y (PMC8660776; doi:10.1038/s41598-021-03178-y)

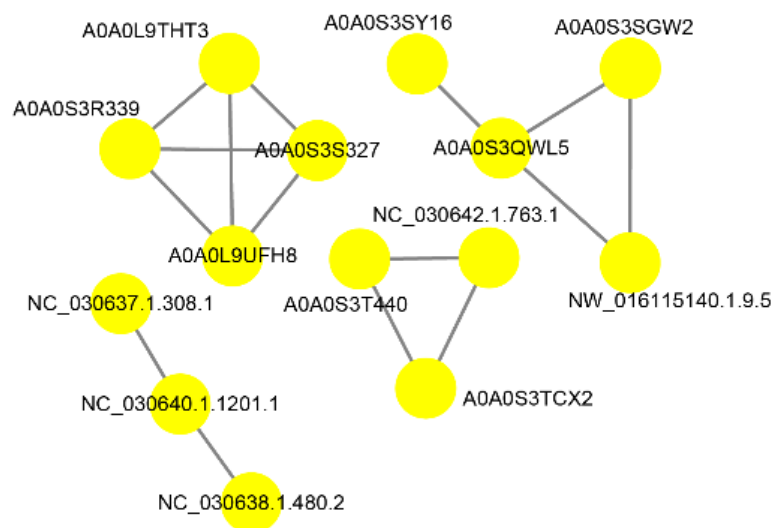

Figure S1 PPI network of differential protein under 17235CK vs 17033CK

Supplement: Supplementary file 1 — Supplementary Information 1. [file 41598_2021_3178_MOESM1_ESM.pdf]
