# Supplementary material for: iTRAQ based protein profile analysis revealed key proteins involved in regulation of drought-tolerance during seed germination in Adzuki bean
Source: Sci Rep. 2021 Dec 9;11:23725. doi: 10.1038/s41598-021-03178-y (PMC8660776; doi:10.1038/s41598-021-03178-y)
Supplement: Supplementary file 2 — Supplementary Information 2. [file 41598_2021_3178_MOESM2_ESM.pptx]

## Slide 1
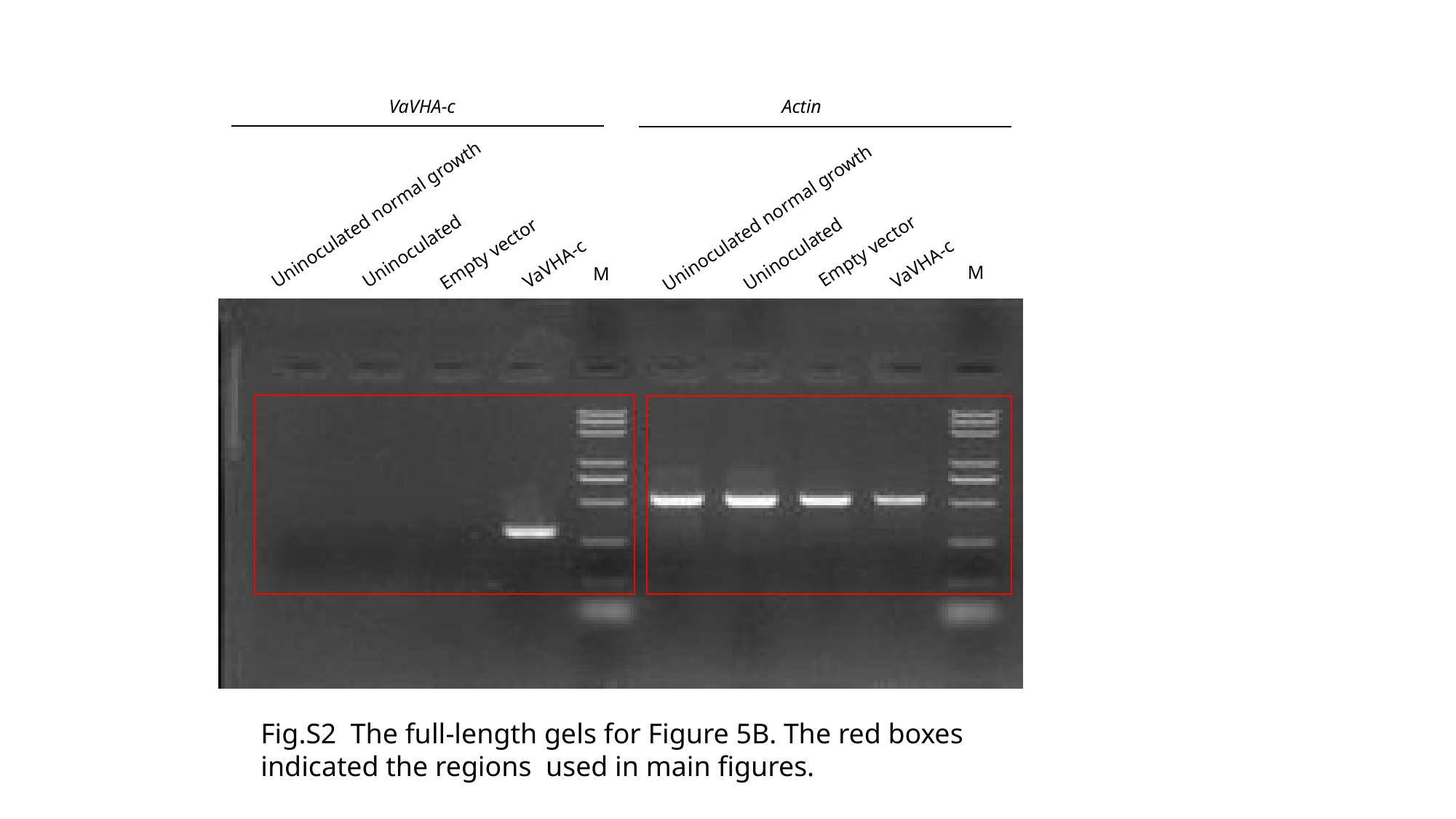

VaVHA-c
Actin
Uninoculated normal growth
Uninoculated normal growth
Uninoculated
VaVHA-c
Uninoculated
Empty vector
Empty vector
VaVHA-c
M
M
Fig.S2 The full-length gels for Figure 5B. The red boxes indicated the regions used in main figures.
